# Genetic Activation of Nrf2 Protects against Fasting-Induced Oxidative Stress in Livers of Mice

**DOI:** 10.1371/journal.pone.0059122

**Published:** 2013-03-18

**Authors:** Yu-Kun Jennifer Zhang, Kai Connie Wu, Curtis D. Klaassen

**Affiliations:** Department of Internal Medicine, University of Kansas Medical Center, Kansas City, Kansas, United States of America; University of Minnesota - Twin Cities, United States of America

## Abstract

Acute fasting causes elevated oxidative stress. The current study investigated the effects of the nuclear factor erythoid 2-related factor 2 (Nrf2), the sensor of oxidative stress in cells, on energy homeostasis and liver pathophysiology during fasting. Feed was removed from mice possessing none (Nrf2-null), normal (wild-type, WT), enhanced (Keap1-knockdown, K1-KD), and maximum (hepatocyte-specific Keap1-knockout, K1-HKO) Nrf2 activity in liver for 24 h. Body weight, blood glucose, and blood lipid profiles were similar among mice with graded Nrf2 activity under either fed or fasted conditions. Fasting reduced liver size in mice expressing Nrf2, but not in Nrf2-null mice. Nrf2-null mice accumulated more non-esterified free fatty acids and triglycerides in liver after fasting than the other genotypes of mice. Fatty acids are mainly catabolized in mitochondria, and Nrf2-null mice had lower mitochondrial content in liver under control feeding conditions, which was further reduced by fasting. In contrast, mitochondrial contents in mice with enhanced Nrf2 activity were not affected by fasting. Oxidative stress, determined by staining of free radicals and quantification of malondialdehyde equivalents, was highest in Nrf2-null and lowest in K1-HKO mice after fasting. The exacerbated oxidative stress in livers of Nrf2-null mice is predicted to lead to damages to mitochondria, and therefore diminished oxidation and increased accumulation of lipids in livers of Nrf2-null mice. In summary, the Nrf2-regulated signaling pathway is critical in protecting mitochondria from oxidative stress during feed deprivation, which ensures efficient utilization of fatty acids in livers of mice.

## Introduction

Starvation is a common situation for living organisms. Complex metabolic systems have evolved to help mammals adapt to food deprivation. The liver plays a central role in maintaining whole-body energy homeostasis during fasting. In the initial stage of fasting, liver glycogen is mobilized to replenish blood glucose (glycogenolysis). When glycogen is depleted, triglycerides stored in adipose tissue are released into the circulation in the form of glycerol and fatty acids. The glycerol is converted into glucose by hepatic gluconeogenesis. The fatty acids are either directly oxidized to generate energy by liver and muscle, or transformed into ketone bodies by the liver. Ketone bodies can be utilized for gluconeogenesis by the liver, or used as fuel for tissues, such as brain, which cannot use fatty acids. Following prolonged fasting, fat reserves are exhausted and muscle degradation occurs to send amino acids to the liver as another substrate for gluconeogenesis [1].

While moderate caloric restriction is beneficial for mammals, acute fasting may be harmful. Livers of rats fasted for 36 h exhibit increased free radicals and decreased anti-oxidant levels [2]. Mitochondrial oxidative phosphorylation produces ATP to support normal cellular function and metabolic homeostasis. However, this process is also the major intracellular source of reactive oxygen species (ROS) or “free radicals”. Free radicals, such as superoxide, hydrogen peroxide, and hydroxyl radicals, are highly reactive molecules that can damage cellular constituents like DNA, proteins, and lipids. Aerobic organisms protect themselves against ROS-induced damage via intrinsic defense systems that include the expression of various antioxidant enzymes (i.e., superoxide dismutase, catalase, and glutathione peroxidase) to convert free radicals into oxygen and water, as well as the maintenance of a significant concentration of reducing equivalents (i.e., reduced glutathione) to neutralize ROS [3].

The cap’n’collar transcription factor Nrf2 (nuclear factor erythoid 2-related factor 2) functions as a major regulator of cellular redox balance [4]. Under quiescent conditions, Nrf2 is retained in the cytoplasm by Kelch-like ECH-associated protein 1 (Keap1) and undergoes constant ubiquitination and proteosomal degradation. When the intracellular oxidative and electrophilic stresses are elevated, Nrf2 is released from Keap1, translocates into the nucleus, and promotes the expression of antioxidant and detoxifying enzymes, such as glutathione *S*-transferase A1 (Gsta1), NADPH quinone oxidoreductase 1 (Nqo1), glutamate cysteine ligase catalytic subunit (Gclc)–the rate-limiting enzyme for glutathione synthesis, as well as the small redox protein thioredoxin (Txn), etc. [5]–[8].

Extensive investigations have been published on the cellular protective roles of Nrf2 against various toxicants and diseases [4], [9], [10]. Recently, the Nrf2-Keap1 signaling pathway has garnered increasing attention in the field of energy metabolism [11]–[16]. Our laboratory has explored the potential role of Nrf2 during the pathophysiological progression of type I diabetes [17], fatty liver disease [18], as well as high-fat diet-induced obesity [19] and glucose intolerance [20] in mice.

Whereas the aforementioned reports investigated the role of the Nrf2-Keap1 system under circumstances of nutrient surplus, the current study explores the potential function of Nrf2 signaling on energy allocation during food deprivation. To achieve this goal, Nrf2-null, wild-type (WT), Keap1-knockdown (K1-KD; enhanced Nrf2 activation in all tissues), and Keap1 hepatocyte-specific-knockout (K1-HKO; maximum Nrf2 activation in the liver and enhanced Nrf2 activation in other tissues) mice [8] were subjected to 24-h fasting. Metabolic parameters in blood and liver as well as expression of genes involved in energy metabolism in liver were determined to elucidate the effects of graded activation of Nrf2 on energy homeostasis under the physiological condition of fasting.

## Materials and Methods

### Animals and Experimental Design

Nrf2 knockout (Nrf2-null) mice were kindly provided by Dr. Jefferson Chan (University of California, Irvine, CA) [21] and Keap1-knockdown (K1-KD) mice were from Dr. Masayuki Yamamoto (Tohoku University, Aoba-ku, Sendai, Japan) [22]. Keap1 hepatocyte-specific knockout (K1-HKO) mice were generated by crossing K1-KD mice and Alb-Cre^+^ mice, which express Cre only in hepatocytes [8]. All strains were backcrossed into more than 99% congenic C57BL/6 background, which were verified by the Jackson Laboratory (Bar Harbor, ME). Wild-type (WT) C57BL/6 mice were purchased from Charles River Laboratories, Inc. (Wilmington, MA). All mice were bred in the Laboratory Animal Facility at the University of Kansas Medical Center and housed in a temperature-, light-, and humidity-controlled environment that is accredited by the Association for Assessment and Accreditation of Laboratory Animal Care. Mice were maintained on laboratory chow (Harlan’s Teklad rodent diet #8604), and had free access to water. Male Nrf2-null, WT, K1-KD, and K1-HKO mice (n = 5, 2-mo old) were either fasted for 24 h (chow was removed from 9∶00 am to 9∶00 am next day) or maintained on the laboratory chow. In a prolonged fasting and refeeding experiment, male mice of the four genotypes (n = 3−5) were either fasted for a continuous 36-h period or fasted for 24 h and refed for 12 h. Animals were euthanized by an overdose of pentobarbital sodium. Plasma and liver were collected and stored at −80°C until use. A portion of liver was embedded in OCT (frozen tissue matrix) followed by flash freezing on dry ice. Another piece of liver was fixed in 10% formalin. The University of Kansas Medical Center Institutional Animal Care and Use Committee approved the studies.

### Histopathology

Liver tissues were fixed in 10% formalin for 48 h, transferred to 70% ethanol for 48 h, and embedded in paraffin blocks for sectioning. Liver sections (5 µm) were stained with hematoxylin and eosin using standard protocols.

### Blood Chemistry

Blood was taken from the tail right before each mouse was euthanized, and glucose concentration was determined using a ReliOn Ultima glucose monitor (Arkray USA, Inc., Minneapolis, MN). Concentrations of triglycerides and nonesterified fatty acids (NEFAs) in plasma were quantified using kits from Wako Diagnostics (Richmond, VA). Beta-hydroxybutyrate concentrations were determined using a kit from Cayman Chemical Company (Ann Arbor, MI) per the manufacturer’s protocol.

### Liver Glycogen and Lipid Assays

Glycogen was prepared from mouse livers as reported [23]. Briefly, 20 mg of frozen liver tissue was homogenized in 0.03 N HCl. The homogenates were heated at 100°C for 5 min, followed by centrifuge at 12,000 g for 5 min. The supernatant was transferred to a new tube and appropriate dilutions were made. Glycogen was hydrolyzed by amyloglucosidase from *Aspergillus niger* (A-7420, Sigma-Aldrich) and the glucose was quantified with a kit (GOGA-20) from Sigma-Aldrich (St. Louis, MO). The assay was carried out according to the manufacturer’s protocol with slight modifications for microplate assays. Liver lipids were extracted as described [24], and quantified similarly as serum lipids.

### Redox Status Assays

Frozen liver tissues embedded in OCT were cut into 6 µM sections and stained with dihydroethidium (DHE) as described previously [25]. Briefly, liver cryosections were stained with 30 µM DHE for 30 min at 37°C, and rinsed 3 times with PBS solution. The oxidation of DHE by intracellular free radicals generates 2-hydroxyethidium and ethidium. The red fluorescence of ethidium-stained DNA was detected using a fluorescent microscope. Photographs of liver sections from various genotypes and treatments were taken using the same contrast and lightness parameters.

GSH concentrations in livers were quantified by UPLC-MS/MS as described previously [8]. Lipid peroxidation was monitored by determining thiobarbituric acid reactive substances (TBARS) as described previously [18]. TBARS was quantified using the OXItek TBARS kit (ZeptoMetrix, Buffalo, NY).

### Mitochondrial DNA Content Assay

The relative copy numbers of mitochondrial DNA (mtDNA) and nuclear DNA (nDNA) in livers were determined by quantitative PCR as described [26]. Each reaction (20 µL) contained genomic DNA (10 ng), SYBR Green reagent (Bio-Rad, Hercules, CA), and forward and reverse primers (0.3 µM each). Specific primers were designed for mitochondrial 16S rRNA gene and nuclear hexokinase 2 (intron 9) gene as described [27]. A standard dilution series was used to confirm the efficiency of exponential amplification for each primer pair.

### RNA Extraction

Total RNA was extracted from tissues using RNA-Bee reagent (Tel-Test, Inc., Friendswood, TX) per the manufacturer’s protocol. RNA was dissolved in diethyl pyrocarbonate-treated deionized water, and RNA concentration was determined with a NanoDrop Spetrophotometer ND-1000 (Thermo Scientific, Wilmington, DE).

### Messenger RNA Quantification

Total RNA was reverse-transcribed into first-strand cDNA using multiscript reverse transcriptase from a High Capacity RT kit (Applied Biosystems, Foster City, CA) according to the manufacturer’s protocol. Messenger RNA of genes of interest was determined with quantitative real time-PCR performed on an Applied Biosystems Prism 7900HT sequence detection system. The reaction system contains 2 ng of cDNA, 150 nM of each primer, and 5 µl of Power SYBR Green PCR Master Mix (Applied Biosystems, Foster City, CA) in a total volume of 10 µl. The specific primers used to quantify gene expression are Nrf2 (forward, 5′-cgagatatacgcaggagaggtaaga-3′; reverse, 5′-gctcgacaatgttctccagctt-3′), Nqo1 (forward, 5′-tatccttccgagtcatctctagca-3′; reverse, 5′-tctgcagcttccagcttcttg-3′), Gsta1 (forward, 5′-cgccaccaaatatgacctct-3′; reverse, 5′-ttgcccaatcatttcagtca-3′), Txn (forward, 5′-gccaaaatggtgaagctgat-3′; reverse, 5′- tgatcattttgcaaggtcca-3′), PGC-1α (forward, 5′- ccgagaattcatggagcaat-3′; reverse, 5′- tttctgtgggtttggtgtga-3′), NRF1 (forward, 5′-agcacggagtgacccaaac-3′; reverse, 5′-tgtacgtggctacatggacct-3′), G6pase (forward, 5′-atgactttgggatccagtcg-3′; reverse, 5′-tggaaccagatgggaaagag-3′), Pepck (forward, 5′–3′; reverse, 5′–3′), PPARα (forward, 5′-atgaagagggctgagcgtag-3′; reverse, 5′-aaacgcaacgtagagtgctgt-3′), Acox1 (forward, 5′-cttggatggtagtccggaga-3′; reverse, 5′-tggcttcgagtgaggaagtt-3′), and Cpt1a (forward, 5′-tgatgacggctatggtgtttc-3′; reverse, 5′-caaacaaggtgataatgtccatc-3′),. The relative mRNA levels were calculated by cycle threshold (Ct) values, which were normalized to the internal control glyceraldehyde 3-phosphate dehydrogenase (Gapdh) mRNA.

### Statistical Analysis

Differences among individual groups were evaluated by two-way analysis of variance (ANOVA) with genotype and treatment as main factors, followed by Student-Newman-Keuls comparisons. Differences were considered statistically significant at *p*<0.05.

## Results

### Body and Liver Weight Changes

**Figure 1:**
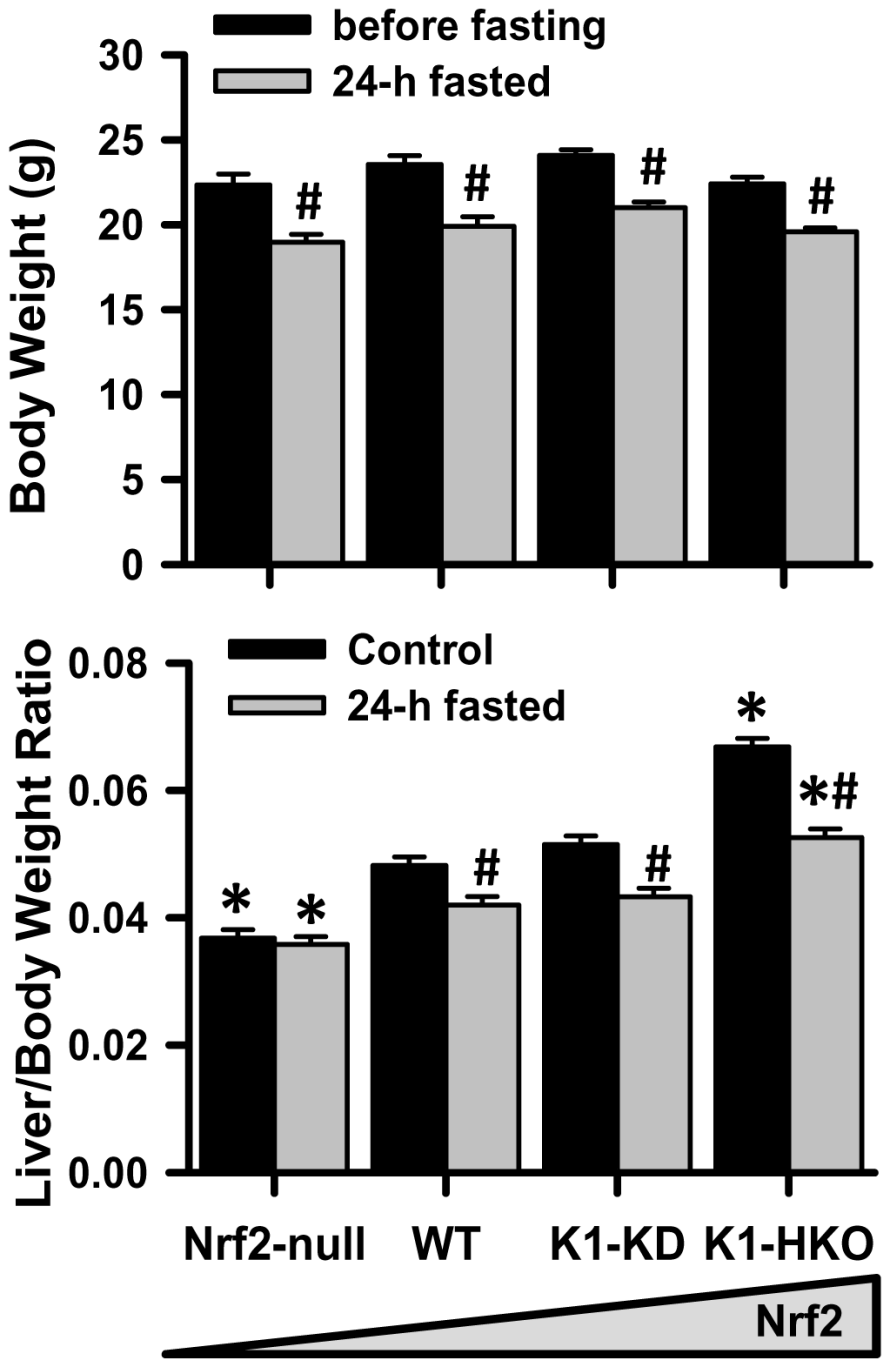
Body weight and liver weight. (A) Body weight. (B) Liver-body weight ratio. Nrf2-null, wild-type (WT), Keap1-knockdown (K1-KD), and Keap1-1 hepatocytes-specific knockout (K1-HKO) mice were either fed laboratory chow or fasted for 24 h. Data are presented as mean ± S.E. (n = 5). Two-way ANOVA was performed with genotype and treatment as main factors. Significant main effects and interactions were followed by Student-Newman-Keuls comparisons to assess the differences between groups. Asterisks (*) represent statistical differences (*p*<0.05) compared with WT mice on the same treatment. Pounds (#) represent statistical differences (*p*<0.05) caused by fasting in (A); and between fasted and control mice of the same genotype in (B).

A 24-h feed deprivation reduced the body weights of mice of all four genotypes (Fig. 1, upper panel). Under control conditions, the liver-to-body weight ratio was lower in Nrf2-null mice, but higher in K1-HKO mice, and not statistically different in K1-KD mice, when compared with WT mice (Fig. 1, lower panel). The 24-h feed deprivation reduced the liver size in WT, K1-KD, and K1-HKO mice, but not in Nrf2-null mice.

### Liver Histopathology

Hematoxylin and eosin staining showed that the lobular architecture and metabolic zonation of the liver were unaffected by a 24-h fasting in all genotypes of mice (data not shown). No apparent genotype differences were observed under either fed or fasted conditions.

### Blood Lipid and Glucose Profiles

**Figure 2:**
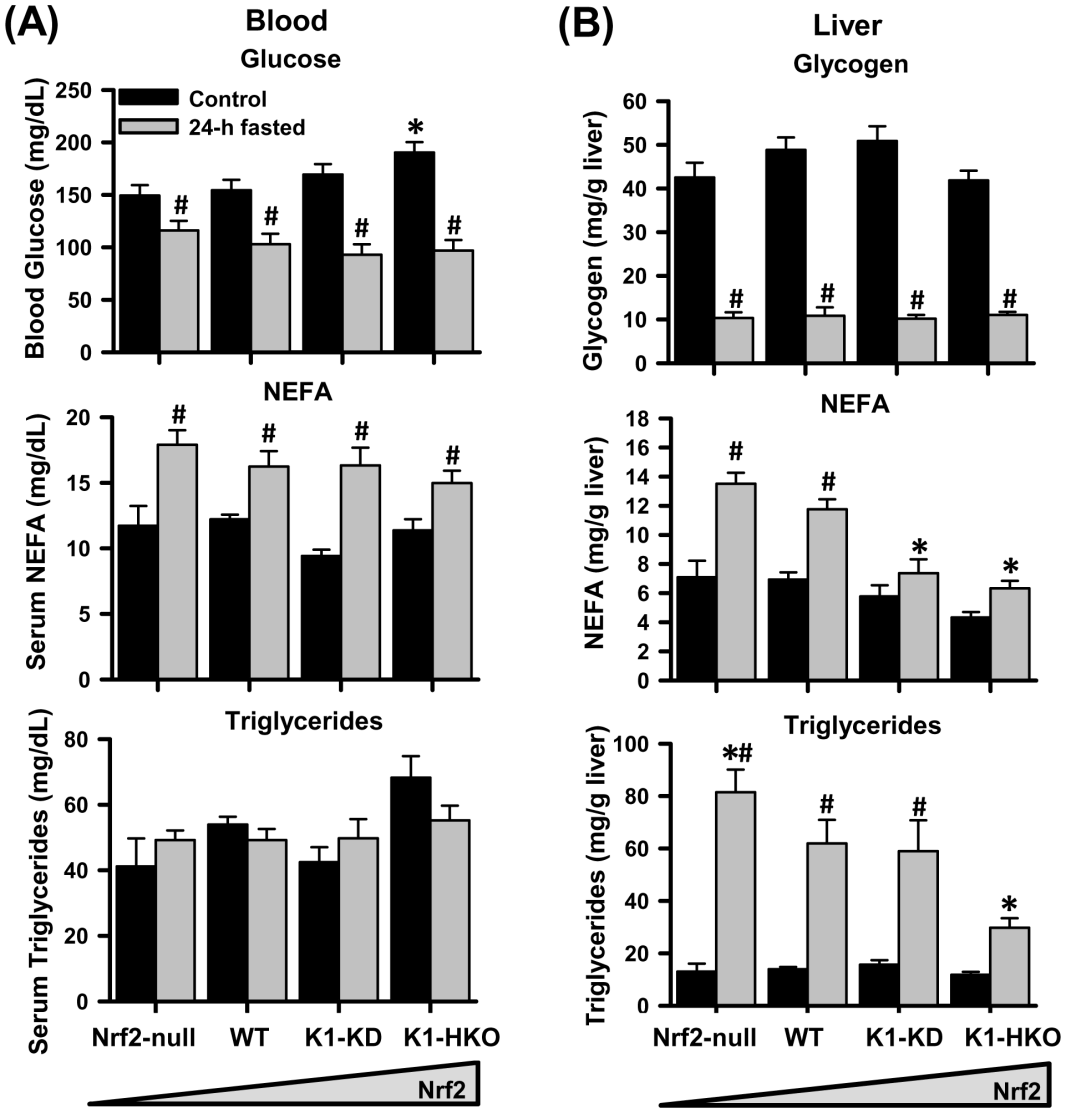
Glucose and lipid profiles in blood and liver. (A) Plasma concentrations of glucose, non-esterified fatty acids (NEFAs), and triglycerides. (B) Liver contents of glycogen, NEFAs, and triglycerides. Data are presented as mean ± S.E. (n = 5). Two-way ANOVA was performed with genotype and treatment as main factors. Significant main effects and interactions were followed by Student-Newman-Keuls comparisons to assess the differences between groups. Asterisks (*) represent statistical differences (*p*<0.05) compared with WT mice on the same treatment. Pounds (#) represent statistical differences (*p*<0.05) between fasted and control mice of the same genotype.

Under control conditions, K1-HKO mice displayed higher blood glucose concentrations than WT mice (Fig. 2A). Feed deprivation reduced blood glucose concentrations in all four genotypes. Conversely, circulating non-esterified fatty acids (NEFAs) were increased in all four genotypes after fasting, and no genotype differences were observed under either control or fasted situations. Serum concentrations of triglycerides were not significantly affected by either fasting or Nrf2 levels in mice. The blood concentrations of β-hydroxybutyrate were markedly elevated by 24-h fasting but was not different among genotypes (data not shown).

### Liver Lipid and Glucose Profiles

The glycogen content in livers was markedly reduced by 24-h feed deprivation (Fig. 2B). However, Nrf2 activity had no effects on glycogen content in livers of mice under either fed or fasted conditions. Whereas hepatic concentrations of NEFAs under the fed condition were similar in all four genotypes of mice, 24-h feed deprivation increased NEFAs concentrations in livers of Nrf2-null and WT mice, but not in the Nrf2-activated K1-KD and K1-HKO mice. The concentration of triglycerides in livers was increased more than 3 fold in Nrf2-null, WT, and K1-KD mice after feed deprivation, but its elevation was not statistically significant in K1-HKO mice. Concentrations of both NEFAs and triglycerides exhibited a trend of being higher in livers of Nrf2-null and lower in K1-HKO mice after fasting.

### Hepatic Expression of Genes Involved in Glucose and Lipid Metabolism

**Figure 3:**
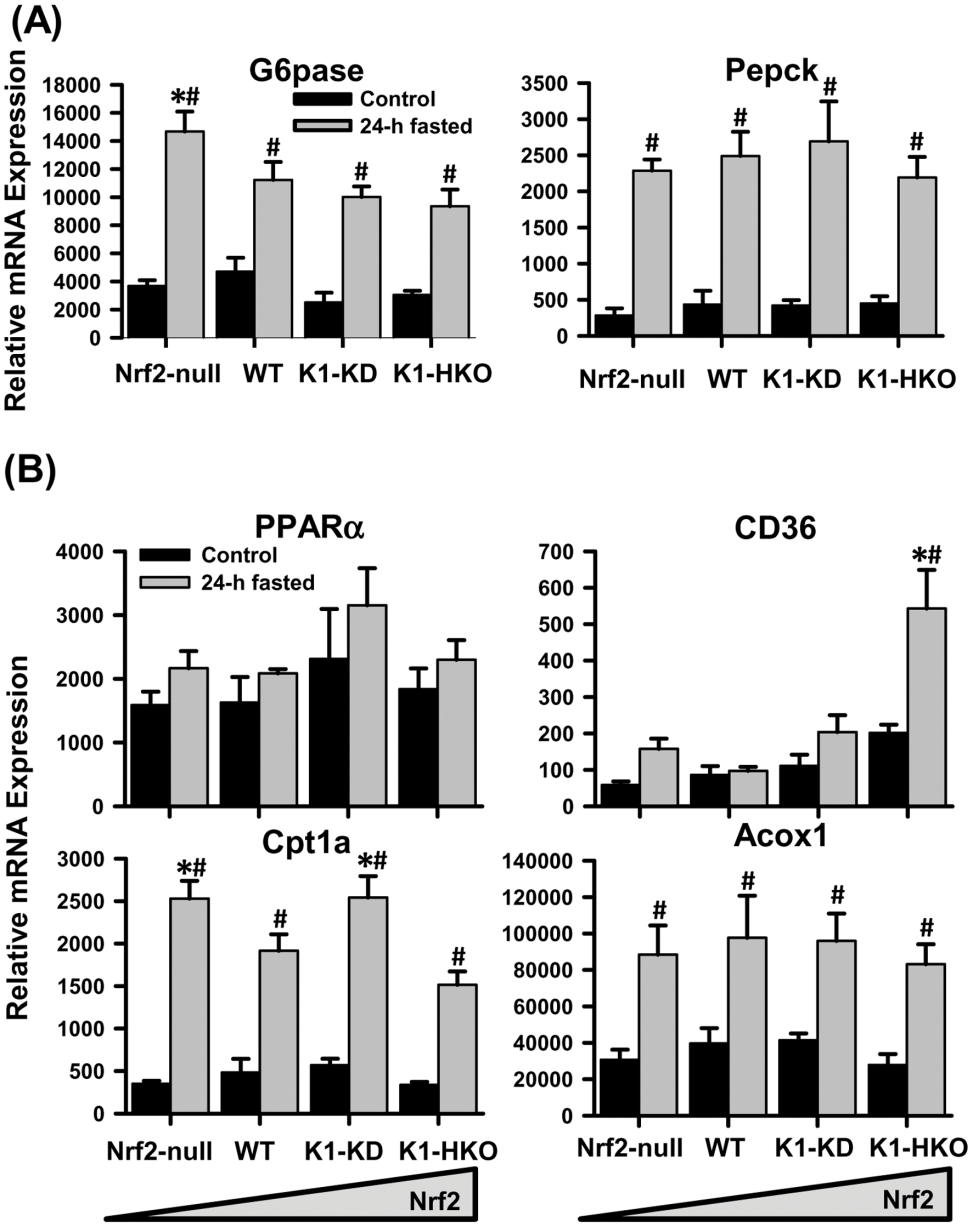
Hepatic expression of genes involved in glucose and lipid metabolism. (A) Messenger RNA of genes encoding key enzymes for gluconeogenesis. (B) Messenger RNA expression of PPARα and genes involved in fatty acid catabolism. Data are presented as mean ± S.E. (n = 5). Two-way ANOVA was performed with genotype and treatment as main factors. Significant main effects and interactions were followed by Student-Newman-Keuls comparisons to assess the differences between groups. Asterisks (*) represent statistical differences (*p*<0.05) compared with WT mice on the same treatment. Pounds (#) represent statistical differences (*p*<0.05) between fasted and control mice of the same genotype.

In order to adapt to food deprivation, the liver produces glucose via enhanced gluconeogenesis to support organs that depend on glucose as the energy source. Glucose-6-phosphatase (G6pase) and phosphoenolpyruvate carboxykinase (Pepck) catalyze two rate-limiting steps of gluconeogenesis in hepatocytes. The expression of these gluconeogenetic genes was not markedly affected by the activity of Nrf2 in *ad libitum* fed mice (Fig. 3A). Fasting increased the mRNA of both enzymes in liver. Messenger RNA of G6pase, but not Pepck, was slightly higher in livers of Nrf2-null mice than other genotypes after fasting. These data indicate that fasting markedly stimulated hepatic gluconeogenesis in mice, which is moderately affected by Nrf2.

Fatty-acid β-oxidation and ketone-body synthesis is another major adaptive response of the liver during the first 24 h of fasting [28]. The peroxisome proliferator-activated receptor alpha (PPARα) is the principal regulator of fatty acid catabolism in liver. PPARα mRNA tended to be induced by 24-h feed deprivation in livers of mice (Fig. 3B), but was not affected by Nrf2 activity under either fed or fasted conditions. The fatty acid translocase CD36, a gene that is regulated by PPARα and several other transcription factors [29], [30], plays a role in both fatty acid uptake by hepatocytes and transport of fatty acids into mitochondria to be oxidized [31]. Under fed conditions, CD36 mRNA tended to be expressed higher in livers of mice with enhanced Nrf2 activity and lower in Nrf2-null mice. Fasting increased CD36 mRNA much more in K1-HKO mice than other genotypes. Mitochondrial oxidation of fatty acids is the major source of ATP during prolonged fasting. Carnitine palmitoyltransferase I (Cpt1) mediates the transport of long-chain fatty acids across the mitochondrial membrane to be oxidized in the mitochondrial matrix. Basal expression of Cpt1a mRNA was not different between genotypes. The mRNA of Cpt1a was induced markedly by 24-h fasting, and its expression was slightly higher in livers of Nrf2-null and K1-KD mice than WT and K1-HKO mice. In addition to mitochondria, peroxisomes also play important roles in oxidizing fatty acids. Messenger RNA of acyl-coenzyme A oxidase 1 (Acox1), the first, and rate-limiting enzyme of peroxisomal β-oxidation, was increased by fasting in livers of all four genotypes of mice. The Acox1 mRNA was not affected by Nrf2 activity under either fed or fasted conditions.

### Fasting-induced Oxidative Stress in Liver

**Figure 4:**
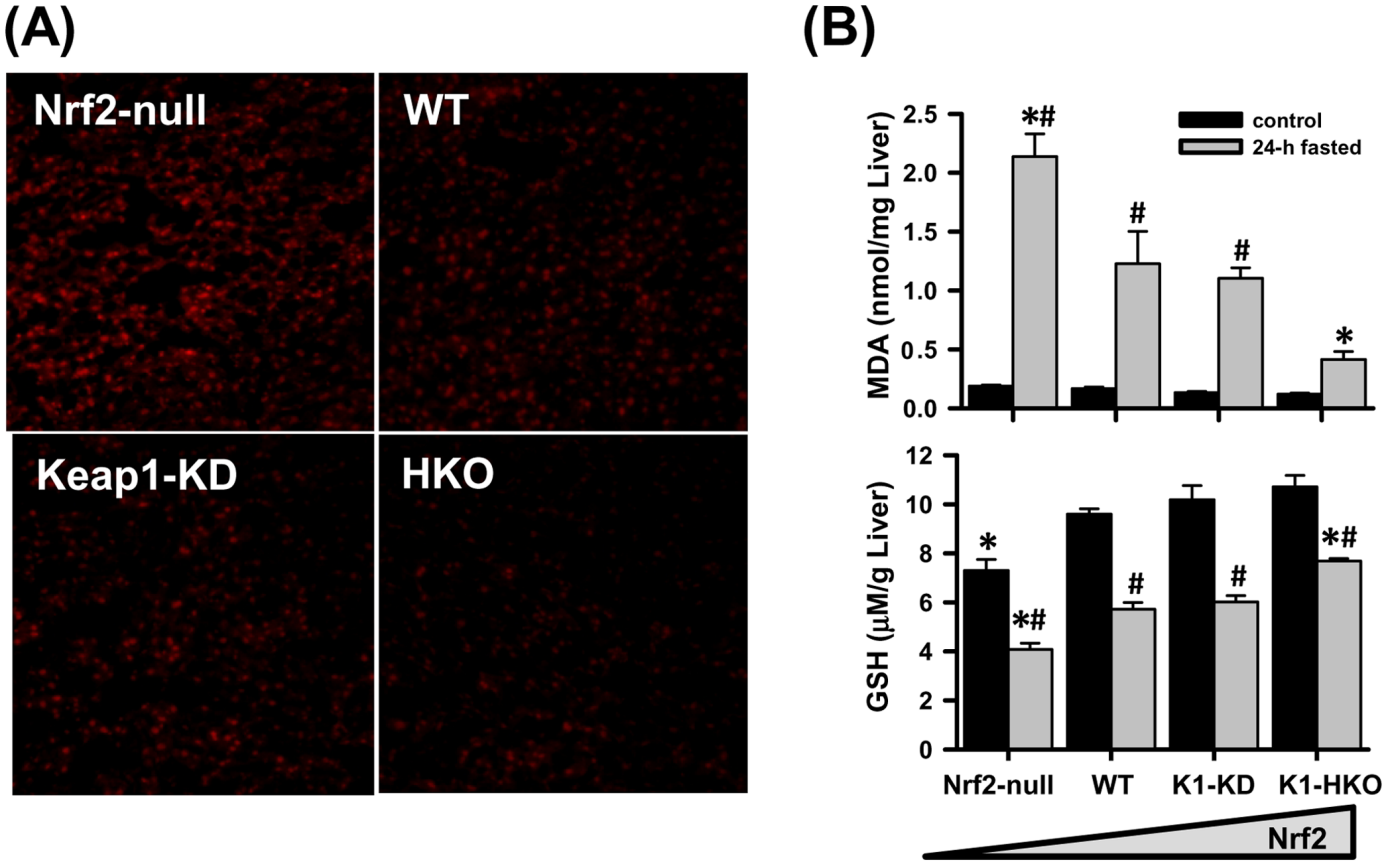
Oxidative stress and glutathione (GSH) contents in mouse livers. (A) Detection of superoxide in liver. Frozen liver tissue was cut into 6 µM sections and stained with dihydrothidium (DHE). (B) Contents of lipid peroxidation products (represented by malonaldehyde equivalent) and the reductive GSH in livers of mice possessing different Nrf2 activity. Data are presented as mean ± S.E. (n = 5). Two-way ANOVA was performed with genotype and treatment as main factors. Significant main effects and interactions were followed by Student-Newman-Keuls comparisons to assess the differences between groups. Asterisks (*) represent statistical differences (*p*<0.05) compared with WT mice on the same treatment. Pounds (#) represent statistical differences (*p*<0.05) between fasted and control mice of the same genotype.

Intracellular oxidative stress induced by fasting was first determined by staining liver cryosections with DHE, a fluorescent probe that detects free radicals and emits red fluorescence. As shown in Fig. 4A, the red fluorescence was strongest in Nrf2-null mice, followed by WT, K1-KD, and weakest in K1-HKO mice, indicating reduced amount of free radicals in liver with enhanced activity of Nrf2 after 24-h fasting. Consistent with the staining results, the content of the lipid peroxidation marker, MDA equivalents, was also markedly elevated by fasting, and was highest in livers of fasted Nrf2-null mice, but to a lower level in fasted WT and K1-KD mice, and not significantly elevated in K1-HKO mice (Fig. 4B). The concentration of the reducing equivalent, GSH, was decreased in livers by fasting in all four genotypes of mice (Fig. 4B). However, Nrf2-null mice had significantly lower hepatic GSH than WT mice under both fed and fasted conditions. In contrast, K1-HKO mice, with the highest Nrf2 activity, tended to have a higher GSH concentration in the liver under control conditions, and retained the highest GSH concentration among the four genotypes after fasting.

### Expression of Nrf2 and Nrf2-target Genes in Liver

**Figure 5:**
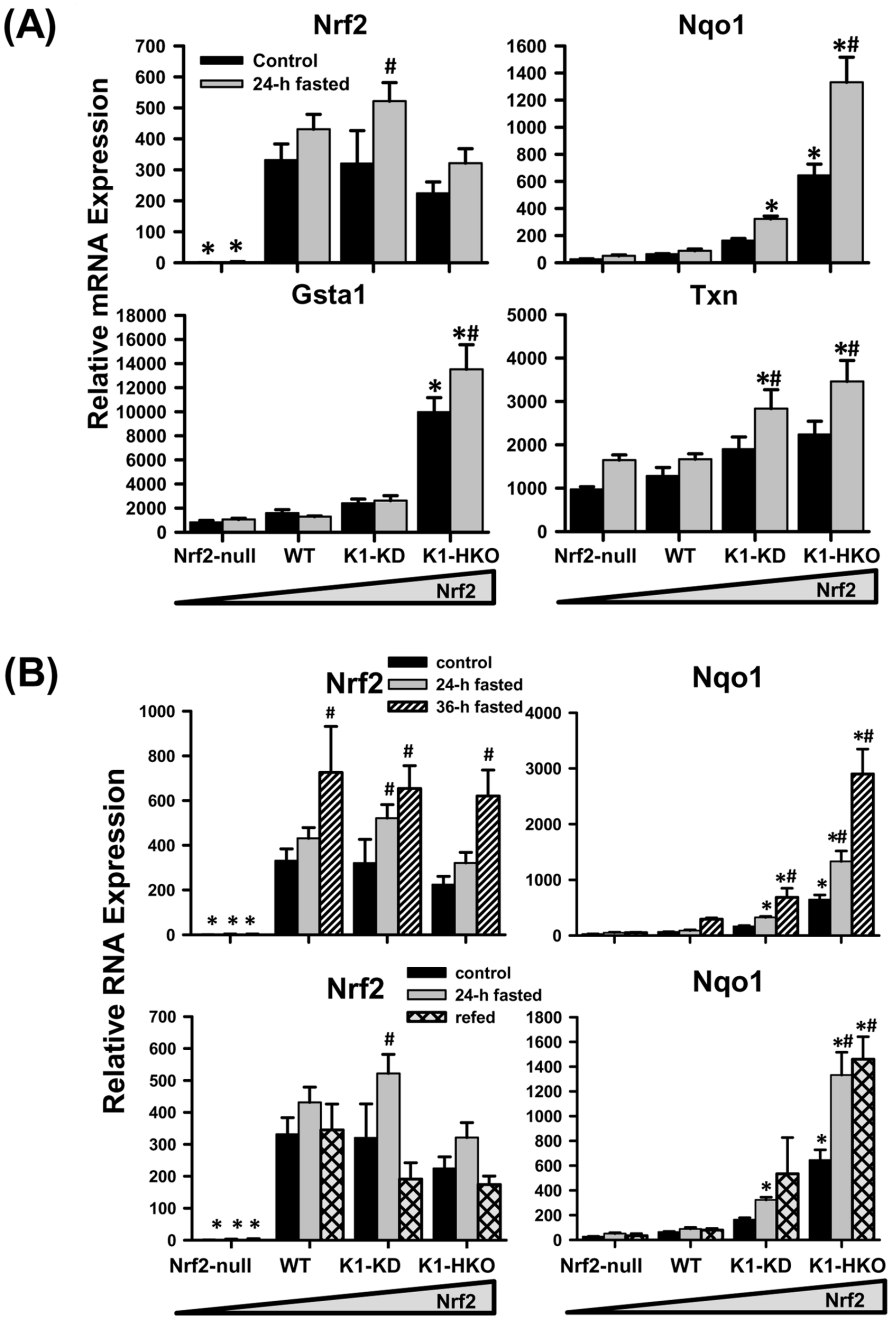
Messenger RNA expression of Nrf2 and Nrf2-regulated genes in mouse livers. (A) Messenger RNA expression of Nrf2 and Nrf2-target genes after a 24-h fasting. (B) Hepatic expression of Nrf2 and Nqo1 after either a 36-h fasting or a 24-h fasting followed by a 12-h refeeding. Data are presented as mean ± S.E. (n = 3−5). Two-way ANOVA was performed with genotype and treatment as main factors. Significant main effects and interactions were followed by Student-Newman-Keuls comparisons to assess the differences between groups. Asterisks (*) represent statistical differences (*p*<0.05) compared with WT mice on the same treatment. Pounds (#) represent statistical differences (*p*<0.05) between fasted (or refed) and control mice of the same genotype.

Hepatic mRNA expression of Nrf2, the master regulator of intracellular redox balance, was induced by 24-h fasting in K1-KD mice (Fig. 5A). The mRNA of several Nrf2-regulated genes that encode detoxification enzymes (Nqo1 and Gsta1) and a redox protein (Txn) were induced by 24-h feed deprivation in K1-HKO mice and/or K1-KD mice, but not in Nrf2-null or WT mice. Under both control and fasted conditions, K1-KD and K1-HKO mice, in which Nrf2 was constitutively activated, expressed or tended to express higher levels of Nqo1, Gsta1, and Txn mRNA than Nrf2-null and WT mice. Moreover, when fasting was extended to 36 h, mRNA of Nrf2 and its prototypical target gene, Nqo1, further increased in livers of WT, K1-KD, and K1-HKO mice, but not in Nrf2-null mice (Fig. 5B, upper panel). Refeeding for 12 h after 24-h fasting decreased Nrf2 mRNA induced by 24-h fasting in K1-KD mice, but not that of Nqo1 in K1-KD or K1-HKO mice (Fig. 5B, lower panel).

### PGC-1α Signaling Pathway and Mitochondrial Content in Liver

**Figure 6:**
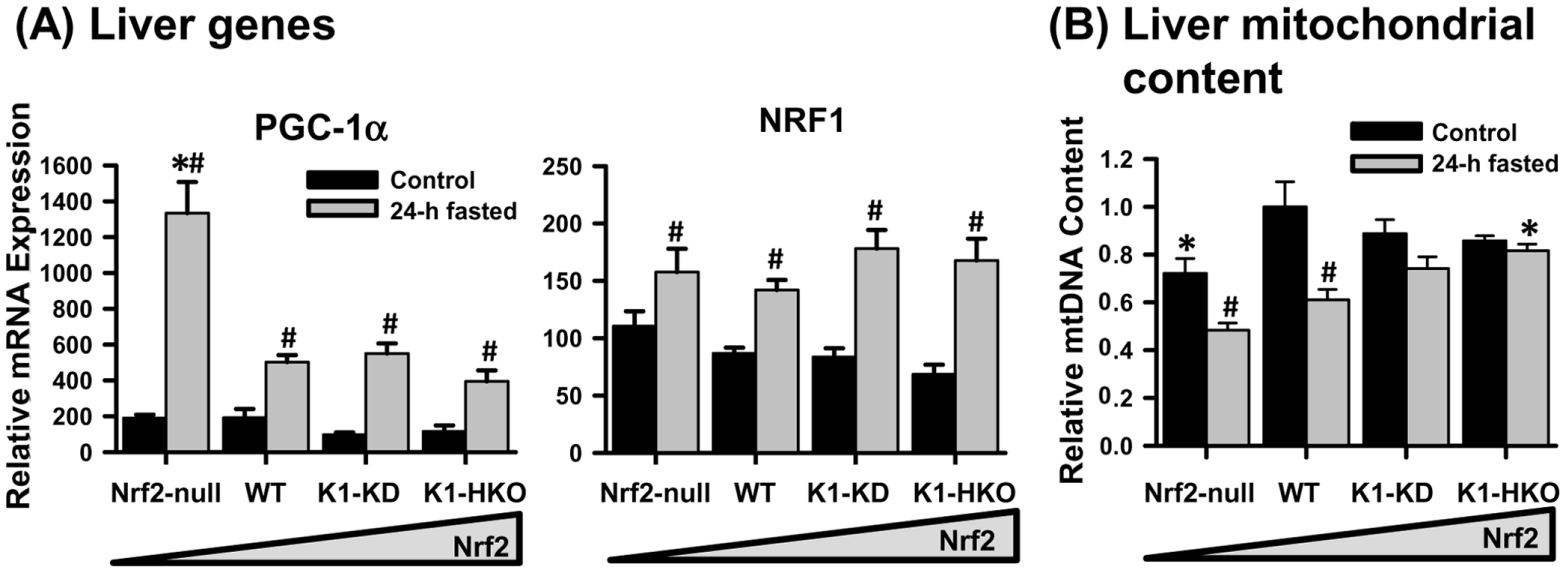
PGC-1α expression and mitochondrial content in livers of mice after 24-h fasting. (A) Messenger RNA expression of transcription factors regulating mitochondria biogenesis in liver. (B) Relative mitochondrial content in livers of control and fasted mice. Data are presented as mean ± S.E. (n = 5). Two-way ANOVA was performed with genotype and treatment as main factors. Significant main effects and interactions were followed by Student-Newman-Keuls comparisons to assess the differences between groups. Asterisks (*) represent statistical differences (*p*<0.05) compared with WT mice on the same treatment. Pounds (#) represent statistical differences (*p*<0.05) between fasted and control mice of the same genotype.

The transcriptional coactivator, peroxisome proliferator-activated receptor gamma coactivator 1-alpha (PGC-1α), plays a critical role in energy metabolism in liver and muscle. Under normal feeding conditions, the PGC-1α mRNA tended to be lower in livers of Nrf2-activated mice than those of Nrf2-null and WT mice. The hepatic expression of PGC-1α mRNA was induced by 24-h fasting in mice of all genotypes, but the induction was significantly higher in livers of Nrf2-null mice than all the Nrf2-expressing mice (Fig. 6A). Nuclear respiratory factor-1 (NRF1) is a transcription factor that is transcriptionally regulated by PGC-1α and plays a key role in mitochondrial biogenesis. The genetic activation of Nrf2 did not affect the basal mRNA expression of NRF1. Fasting induced hepatic expression of NRF1, which was not influenced by the graded Nrf2 activity in mice. Moreover, the hepatic mitochondrial content, determined by the relative mitochondrial DNA to nuclear DNA ratio, in Nrf2-null mice was lower than in WT mice when they were fed *ad libitum* (Fig. 6B). The feed deprivation reduced hepatic mitochondrial contents in Nrf2-null and WT mice, but not in K1-KD and K1-HKO mice.

## Discussion

Nrf2, the master regulator of cellular redox balance, recently garnered increasing attention in the field of nutrient metabolism. Several research groups have investigated the effects of Nrf2 activation on energy homeostasis and the development of diabetes in mice fed a high-fat diet [11]–[20]. In contrast to the energy-surplus condition created by consuming high-calorie diets, fasting reflects a situation that requires an animal to mobilize stored nutrients and convert them into energy to support the function of vital organs. The current study investigated the effects of genetic ablation or activation of Nrf2 on energy homeostasis in mice that were deprived of feed for 24 h. Because the liver plays a central role in fasting physiology [1], energy metabolism in this organ is the emphasis of the current study.

A previous time-course transcriptomic study of fasting (0 to 72 h) demonstrates that the adaptive responses of mouse livers, including fatty acid β-oxidation, ketone biosynthesis, gluconeogenesis, and amino acid oxidation, peaks around 24 h after the onset of food deprivation [28]. Thus, in the current study, mice with none to maximum hepatic activity of Nrf2 were fasted for 24 h. The body weights mice with graded Nrf2 activity were similar after a 24-h fasting (Fig. 1). Furthermore, the fasted serum parameters, including glucose, free fatty acids, triglycerides (Fig. 2A), and ketone bodies in the form of β-hydroxybutyrate (data not shown) were also comparable among the four genotypes of mice. These data suggest that whole-body energy utilization during fasting was not significantly affected by Nrf2. However, 24-h fasting exerted different effects on liver size of mice with graded Nrf2 activity. The liver-to-body weight ratio was decreased by fasting in Nrf2-expressing mice, but remained unaffected in Nrf2-null mice. This phenomenon indicates that the hepatic metabolism of certain nutrients, such as glycogen, fatty acids, and amino acids, during fasting might be altered due to Nrf2 deficiency. The hepatic glycogen content (Fig. 2B) and expression of gluconeogenesis genes (Fig. 3A) were not affected by Nrf2 activity, suggesting Nrf2 has negligible effects on glycogen storage, glycogenolysis, and gluconeogenesis functions of the liver during fasting. In contrast, concentrations of non-esterified free fatty acids and triglycerides were higher in livers of fasted Nrf2-null mice, but lower in Nrf2-activated K1-HKO and/or K1-KD mice than in WT mice. The higher lipid content in livers of fasted Nrf2-null mice could, at least, partially explain why the liver-to-body weight ratio was not affected by fasting in these mice.

Both enhanced uptake of free fatty acids from the blood stream and reduced biotransformation of fatty acids within hepatocytes could lead to increased retention of lipids in liver. The fatty acid translocase (CD36) is an important protein for fatty acid transport in muscle, heart, and liver. CD36 is located on both plasma and mitochondrial membranes, where it facilitates the uptake of fatty acids into hepatocytes, and transports fatty acids into mitochondria to be oxidized, respectively [31]. The CD36 mRNA tended to be increased by fasting in Nrf2-null mice, but significant induction was observed only in K1-HKO mice (Fig. 3B). Thus, the higher lipid content in livers of fasted Nrf2-null mice is unlikely due to increased hepatic uptake of fatty acids. The marked induction of CD36 in livers of fasted K1-HKO mice, on the contrary, might contribute to enhanced mitochondrial fatty acid oxidation, and thus less lipid accumulation in their livers. Moreover, Nrf2 was reported to directly regulate the transcription of the CD36 gene in macrophages [32], which could also be the case in hepatocytes. The mRNA of other enzymes involved in mitochondrial β-oxidation (i.e., Cpt1a) or peroxisomal β-oxidation (i.e., Acox1) were increased in livers of all fasted mice in an Nrf2-independent manner (Fig. 3B). Moreover, 24-h fasting increased the nuclear accumulation of PPARα protein, the primary regulator of fatty acid catabolism, to a similar extent in mice of all genotypes (data not shown). Collectively, these data indicate that, in addition to PPARα, activation of Nrf2 facilitates fatty acid catabolism in livers of fasted mice probably through its positive effects on mitochondrial CD36.

Although the expression profile of CD36 provides some clue, the exact mechanism through which Nrf2 affects lipid metabolism in fasted livers is not clear. One promising theory is that the deficiency or activation of Nrf2 alters the redox status of the cell, which in turn will affect cellular processes that require either reducing equivalents or ROS signaling. Fasting is known to cause increased production of free radicals and suppressed expression of antioxidant enzymes in rodent livers [2], which is also observed in the current study (Figs. 4A and 4B). The present study further shows that this increased oxidative stress posed by 24-h fasting activated the Nrf2 signaling pathway, and resulted in the induction of prototypical Nrf2 target genes in mice with enhanced Nrf2 activity (Fig. 5A). In fact, mRNAs of both Nrf2 and Nrf2 target gene, Nqo1, were induced by prolonged fasting (36-h) in livers of WT, K1-KD, and K1-HKO mice, but not in Nrf2-null mice (Fig. 5B). The activation of Nrf2 during fasting and the subsequent induction of multiple antioxidant and detoxifying enzymes ensure a less oxidative cellular environment in livers of mice expressing Nrf2, but this protection is absent in Nrf2-null mice (Figs. 4A and 4B).

Mitochondria convert acetate derived from carbohydrates, lipids, and proteins into the ultimate energy ATP. During this process, mitochondria also produce a significant amount of free radicals, which results in various cellular injuries, especially to mitochondria themselves. Theoretically, a cell that is well-equipped with antioxidant system is more efficient in energy production when it has more mitochondria. This is the case in the hepatocytes of Nrf2-activated K1-KD and especially the K1-HKO mice, which exhibited less oxidative stress, maintained their mitochondrial content during fasting (Fig. 6B), and retained lower amounts of non-esterified free fatty acids and/or triglycerides (Fig. 2B). In contrast, in livers of fasted Nrf2-mice, in which the Nrf2-regulated oxidative stress-responding system was absent, oxidative stress was elevated, the mitochondrial content was lower, and more lipids accumulated.

Activation of Nrf2 protects mitochondria under various circumstances. For example, the activation of Nrf2 by sulforaphane increased the antioxidant levels in hepatic mitochondria and protected against mitochondrial permeability transition pore opening [33]. In livers and hippocampus of rats, Nrf2 activation and expression of Nrf2-regulated cellular protective genes are essential in maintaining the mitochondrial redox state under mild oxidative stress caused by a ketogenic diet [34]. The activation of Nrf2 actually enhances mitochondrial biogenesis in rat hippocampal neurons by promoting transcription of NRF1 [35]. In the current study, fasting induced NRF1 mRNA livers of mice, but the induction was not affected by Nrf2 activity. PGC-1α plays a key role in regulating the transcription of NRF1 as well as mitochondrial biogenesis. PGC-1α mRNA was induced more by fasting in livers of Nrf2-null mice, which have the least mitochondria under either fed or fasted conditions, than Nrf2-expressing mice (Fig. 6). Thus, the preserved mitochondrial content in livers of fasted K1-KD and K1-HKO mice, which have enhanced activation of Nrf2, appears to be due to the protection provided by the more reduced cellular environment, instead of increased mitochondrial biogenesis.

In summary, Nrf2 activation facilitates lipid catabolism in mouse livers during fasting, but did not significantly affect body. Nrf2 could influence fatty acid metabolism in liver through its direct regulation of fatty acid metabolism-related genes, such as CD36. Moreover, the activation of Nrf2-regulated antioxidant signaling during fasting protects mitochondria from oxidative damages, which ensures efficient fatty acid catabolism in the liver.
